# Supplementary material for: The Effect of Endurance Training on Pulmonary V˙O2 Kinetics in Solid Organs Transplanted Recipients
Source: Int J Environ Res Public Health. 2022 Jul 26;19(15):9097. doi: 10.3390/ijerph19159097 (PMC9331393; doi:10.3390/ijerph19159097)
Supplement: Supplementary file 1 [file ijerph-19-09097-s001.zip › ijerph-1725923-supplementary.pdf]

Table S1. Pulmonary O_2 uptake ($\dot{V}O_{2p}$) and heart rate (HR) kinetics parameters assessed during double leg moderate constant load exercise (DL-MOD) before (PRE) the endurance training period of the heart transplant (HTx) and non-cardiac transplant recipients (kidney and liver transplanted patients).

$\dot{V}O_{2p}$ kinetics	HTx (n = 11)	Non-cardiac transplant recipients (n = 18)	P
O2 Def (mL O2)	804 ± 183	680 ± 244	0.160
MRT (sec)	55.8 ± 15.5	50.4 ± 14.8	0.353
* SCamp (mL O2)	214 ± 69	202 ± 73	0.629
**HR kinetics**	**HTx (n = 10)**	**Non-cardiac transplant recipients (n = 17)**	
Baseline	80.1 ± 15.1	74.8 ± 9.3	0.267
Amplitude	21.1 ± 6	25.4 ± 6.3	0.095
Time delay (sec)	20.3 ± 15.3	7.5 ± 6.4	0.005
Time constant (sec)	118.3 ± 79.5	42.4 ± 18.4	0.001
MRT (sec)	138.6 ± 88.1	49.9 ± 20.3	<0.001
95% CI for time constant	87 - 112	36 - 49	-

Values are expressed as mean ± standard deviation, note that $\dot{V}O_{2p}$ slow component amplitude (SCamp) refers to double leg heavy constant load exercise. *: HTx (n = 13); non-cardiac transplant recipients (n = 20).

Table S2. Pulmonary O_2 uptake ($\dot{V}O_{2p}$) and heart rate (HR) kinetics parameters assessed during double leg moderate constant load exercise (DL-MOD) before (PRE) the endurance training period of the patients taking β-blockade medications and the patients not undergoing β-blockade therapy.

$\dot{V}O_{2p}$ kinetics	β-blockade (n= 12)	Not β-blockade (n= 17)	P
O2 Def (mL O2)	676 ± 157	711 ± 240	0.672
MRT (sec)	48.1 ± 11.8	51.2 ± 14.3	0.556
* SC_{amp} (mL O2)	219 ± 76	197 ± 66	0.370
**HR kinetics**	**β-blockade (n= 10)**	**Not β-blockade (n= 17)**	**P**
Baseline	69.7 ± 7.8	80.6 ± 12.3	0.019
Amplitude	21.9 ± 4.9	22.5 ± 7.6	0.193
Time delay (sec)	16.3 ± 16.2	10.3 ± 8.9	0.228
Time constant (sec)	71.2 ± 47.1	69.8 ± 68.7	0.955
MRT (sec)	87.5 ± 62.1	80.2 ± 74.1	0.795
95% CI for time constant	55 - 66	55 - 76	-

Values are expressed as mean ± standard deviation, note that $\dot{V}O_{2p}$ slow component amplitude (SC_{amp}) refers to double leg heavy constant load exercise. *: β-blockade (n = 15); Not β-blockade (n = 18).
